# Interannual Bacterial Diversity Variability in Antarctic Snow/Ice Samples in the Vicinity of Concordia Station

**DOI:** 10.3390/life16040596

**Published:** 2026-04-03

**Authors:** Gerardo A. Stoppiello, Ricardo Belmonte-Lopes, Caterina Ripa, Daniela Billi, Laura Selbmann

**Affiliations:** 1 Department of Ecological and Biological Sciences, University of Tuscia, 01100 Viterbo, Italy; ricardo.lopes@unitus.it (R.B.-L.); cripa@unitus.it (C.R.); selbmann@unitus.it (L.S.); 2 Department of Biology, University of Rome Tor Vergata, 00133 Rome, Italy; billi@uniroma2.it; 3 Mycological Section, Italian Antarctic National Museum (MNA), 16121 Genoa, Italy; 4 Department of Earth Systems Science and Environmental Technologies (CNR-ISP), CNR-Institute of Polar Sciences, 98122 Messina, Italy

**Keywords:** Antarctic Plateau, bacterial assemblages, ice/snow

## Abstract

In this study, we compared the bacterial diversity of two independent snow and ice sampling campaigns conducted in 2015–2016 and 2018–2019 at Dome C, Concordia Station, Antarctica. Using 16S rRNA gene amplicon sequencing, we analysed 81 samples and, after quality filtering and rarefaction, obtained approximately 3.8 million high-quality reads. Alpha diversity analyses revealed comparable richness between the two sampling periods, while community evenness was higher in 2018–2019. In contrast, all beta diversity metrics consistently showed significant differences in community composition between years, while beta dispersion analyses indicated distinct levels of heterogeneity within the year. The results of the Raup-Crick null model (R0) analyses showed that the observed differences did not deviate from random expectations under the applied null hypothesis. Overall, these results indicate pronounced interannual variability in bacterial assemblages at Concordia Station and suggest that temporal changes in community composition are consistent with assembly processes dominated by episodic inputs and limited persistence under extreme environmental conditions. This study implements previous investigations by providing a comparative temporal perspective and contributes to a better understanding of microbial dynamics in one of the most isolated and low-biomass environments on Earth.

## 1. Introduction

Antarctica is one of the most isolated and extreme environments on Earth. Biological inputs are scarce here and mainly originated from long-range atmospheric transport or sporadic human activities. Airborne microorganisms play a key role in dispersing microbial life to remote, oligotrophic ecosystems, including polar regions, where atmospheric deposition is a significant source of microbial input in snow and ice environments [1,2,3]. In the inland regions of the Antarctic continent, however, katabatic winds flow from the polar plateau towards the coast. These strong, persistent winds may limit the local movement and redistribution of airborne propagules, potentially reducing microbial exchange between inland environments. The French-Italian Concordia research station (75°06′ S, 123°23′ E; 3233 m above sea level) is located on Dome C on the Antarctic Plateau and represents a unique natural laboratory for studying the diversity, dispersal and survival of microbes under extreme environmental conditions. The area is characterised by permanently sub-zero temperatures, intense ultraviolet solar radiation, severe dryness and extremely low nutrient availability. Such conditions exert strong selective pressure on microorganisms, permitting the survival of only those capable of tolerating multiple environmental stresses [4,5,6]. Therefore, the Concordia area has been proposed as a valuable terrestrial analogue for frozen extraterrestrial environments, offering important insights into microbial persistence in ultra-oligotrophic and low-biomass systems, as well as into contamination risks in highly controlled environments [7]. Prior research has demonstrated the presence of detectable microbial assemblages in the surface snow and ice around Concordia Station, despite extremely low biomass levels. These assemblages are primarily composed of bacteria that have been transported through atmospheric deposition or introduced through local human activities [8,9]. Napoli et al. (2022) provided the first description of bacterial diversity in this environment based on 16S rRNA gene amplicon sequencing. This demonstrated that high-throughput sequencing approaches can successfully detect microbial signatures even when DNA concentrations are close to the limits of analytical detection [8]. More recently, Stoppiello et al. (2023) refined sampling strategies, investigating bacterial and fungal diversity at various distances from the station and across different seasons. This study emphasised the transient and highly variable nature of microbial assemblages in this environment [9]. Despite these advances, the temporal variability of bacterial assemblages at Concordia Station has not yet been investigated through direct comparisons between different sampling years. In environments with extremely low biomass, such as Antarctic Plateau snow, microbial assemblages may not represent stable, structured communities, but rather stochastic assemblages resulting from episodic atmospheric deposition events [10,11]. It is important to understand whether the observed variability reflects random annual inputs or more persistent environmental influences when interpreting microbial diversity patterns in polar cryospheric habitats. In this study, we compared bacterial assemblages obtained from two independent sampling campaigns of snow and ice conducted at Concordia Station during the 2015–2016 and 2018–2019 austral summers. Therefore, our aim was to evaluate interannual variability in bacterial assemblages at the same sampling site, and to determine whether detectable differences in microbial composition occur over time in one of the most extreme and isolated microbial habitats on Earth.

## 2. Materials and Methods

### 2.1. Dataset Acquisition, Processing and Location

**Figure 1:**
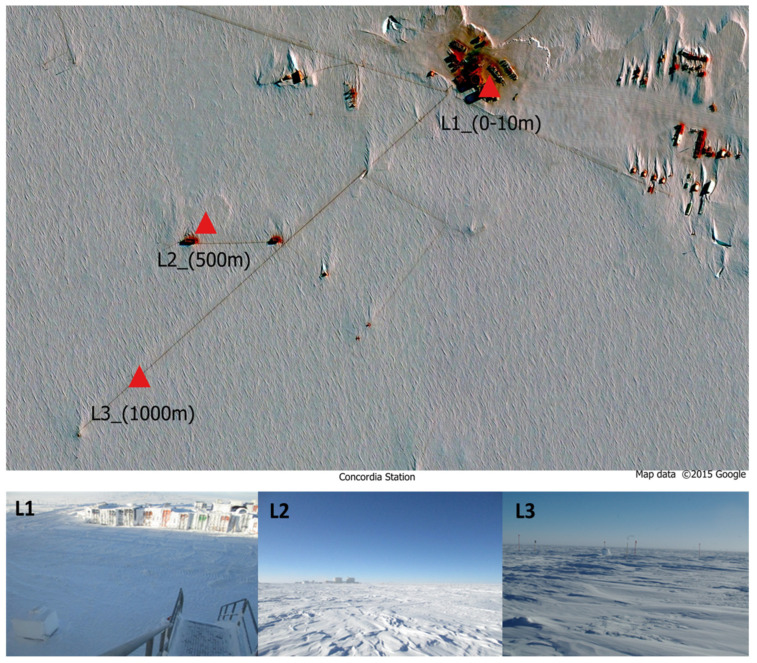
Map of the three sampling sites at different distances from Concordia Station. Area L1, just outside the building; area L2, 500 m distance; area L3, 1000 m distance.

Bacterial amplicon-sequencing datasets from snow and ice samples were retrieved from the public domain. Raw sequencing data were downloaded from the NCBI Sequence Read Archive (SRA) using the SRA Toolkit [12]. All datasets originate from sampling campaigns designed, conducted, and published by the same research consortium, ensuring consistency in sampling strategy, site selection, and study objectives. A total of 81 samples were included in the present analysis: 39 samples collected during the 2018–2019 sampling campaign at the Italian-French Concordia Station (Stoppiello et al., 2023) [9], and 42 samples derived from a comparable survey conducted in 2015–2016 (Napoli et al., 2022) [8]. For the purposes of this study, samples were aggregated by year of collection. This approach was adopted to enable a direct interannual comparison between two independent sampling campaigns, rather than to resolve seasonal or short-term variability, which has already been addressed in detail in previous studies [8,9]. All samples considered here were collected from the same predefined sampling areas located at three progressive distances from the Concordia Station (75°06′01.8″ S, 123°21′03.8″ E) (Figure 1). The Concordia Station is located at Dome C on the Antarctic Plateau, at an altitude of 3233 m above sea level. Air temperatures can reach −80 °C during winter, with an annual mean of approximately −50 °C. The closest permanently inhabited research station is Vostok, located about 600 km away. Concordia hosts up to 80 people during the austral summer and a reduced overwintering crew of approximately 13 personnel, representing one of the most isolated human settlements on Earth.

### 2.2. Bioinformatics

All samples were screened for potential extraction contaminants using the *decontam* R package 4.2.2 [13], and only uncontaminated samples were retained for downstream analyses. Raw paired-end Illumina reads targeting the bacterial 16S rRNA gene were processed using the AMPtk pipeline (v1.2.1) [14]. Sequences were trimmed to a uniform length of 250 bp, and reads shorter than 100 bp were removed. Chimeric sequences were identified and removed using USEARCH (v9.2.64) with default parameters [15,16]. Sequence quality filtering was performed using an expected error threshold of <1.0. Amplicon sequence variants (ASVs) were inferred using a DADA2-based denoising approach [17]. Low-abundance ASVs, defined as singletons or ASVs represented by fewer than five reads across the entire dataset, were excluded from subsequent analyses to reduce potential noise. Taxonomic assignment was performed using the Ribosomal Database Project (RDP) Classifier, version 11.5 (released in 2023) [18], through the hybrid SINTAX/UTAX algorithm implemented in AMPtk [14]. Non-bacterial and unclassified ASVs were removed prior to statistical analyses. Although the two original studies employed different DNA extraction protocols, both were optimised for low-biomass Antarctic samples and followed stringent contamination control procedures [8,9]. In the present work, all sequence data were reprocessed through a single unified bioinformatic pipeline to minimise analytical biases and ensure comparability between datasets.

### 2.3. Biodiversity Indexes and Statistical Analyses

From the raw data, 6,671,457 reads were obtained. After quality filtering and rarefaction to the minimum library size (47,439 reads/sample), 3,842,559 reads remained. Analyses were performed in R v4.3.2, using phyloseq [19], microeco [20], and vegan [21]. Alpha diversity indices included Observed richness, Chao1, and Shannon. Differences between years were tested using the Wilcoxon rank-sum test. Beta diversity was assessed using Bray–Curtis, Jaccard, Weighted UniFrac and Unweighted UniFrac. Ordination was performed via PCoA. Differences in community composition were tested with PERMANOVA (NPMANOVA) with 999 permutations [22]. Within-group heterogeneity (beta dispersion) was calculated using *cal_group_distance()* in microeco. To distinguish stochastic vs. deterministic assembly processes, a Raup-Crick null model was applied using the *ecosimu()* function in vegan with the R0 randomization scheme [23]. All figures were generated using ggplot2 [24]. Significance thresholds were set at *p* < 0.05 unless otherwise specified.

## 3. Results

### 3.1. Bacterial Assemblages’ Composition

**Figure 2:**
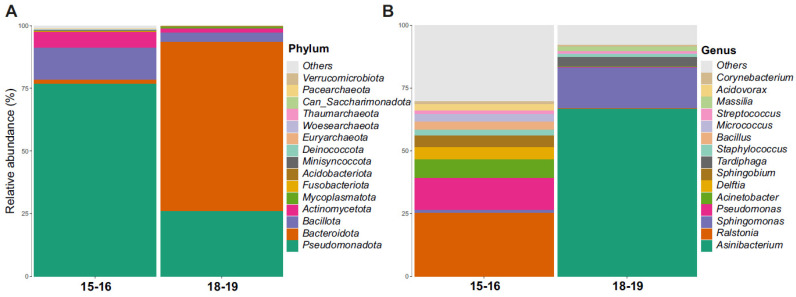
Relative abundance of bacterial taxa at the phylum (**A**) and genus (**B**) levels in samples collected during the two sampling years (2015–2016 and 2018–2019). Bar plots show the mean relative abundance of the dominant taxa within each year. The two assemblages display markedly distinct bacterial compositions.

The bacterial communities identified in the snow and ice samples collected around Concordia Station showed notable differences between the two sampling years (Figure 2A,B).

At the phylum level, the 2015–2016 assemblages were dominated by *Pseudomonadota* (76.8%), followed by *Bacillota* (12.8%) and *Actinomycetota* (6.2%). Minor fractions were represented by *Bacteroidota* (1.5%) and unclassified taxa (1.2%). In contrast, the 2018–2019 samples were dominated by *Bacteroidota* (67.5%), followed by *Pseudomonadota* (26.0%), *Bacillota* (3.7%) and *Actinomycetota* (1.4%), with a small proportion of unclassified sequences (<1%). At the genus level, in 2015–2016 the most abundant taxa were *Ralstonia* (25.2%), *Pseudomonas* (12.7%), *Acinetobacter* (7.2%), *Delftia* (5.0%), and *Sphingobium* (4.7%), together with *Bacillus* (3.4%) and *Micrococcus* (2.8%). Unclassified genera accounted for approximately 7.3% of the total community. In 2018–2019, the assemblages were dominated by *Asinibacterium* (66.7%), followed by *Sphingomonas* (16.2%) and *Tardiphaga* (3.7%), while the remaining genera contributed marginally to the overall composition.

The two temporal assemblages exhibited markedly distinct bacterial profiles, with limited taxonomic overlap across the two years.

### 3.2. Alpha Diversity

**Figure 3:**
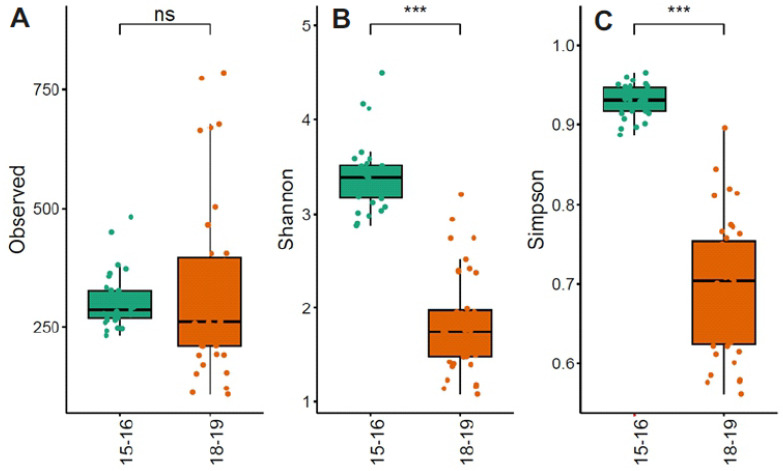
Comparison of alpha diversity using Observed, Shannon, and Simpson indexes of bacterial communities (**A**–**C**) between the two sampling years (2015–2016 and 2018–2019). Statistical support was tested by the Wilcoxon rank-sum test and is indicated as *** *p* < 0.001; ns = not statistically significant.

Alpha diversity indices revealed clear differences between the two sampling years (Figure 3A–C).

The Observed richness showed comparable values between 2015–2016 and 2018–2019, with no significant difference detected (Wilcoxon rank-sum test, *p* = 0.44).

Conversely, both the Shannon and Simpson diversity indices were significantly higher in the 2018–2019 samples compared to those from 2015–2016 (Wilcoxon rank-sum test, *p* < 0.001).

All statistical results are detailed in Supplementary Materials Table S1.

### 3.3. Beta Diversity

**Figure 4:**
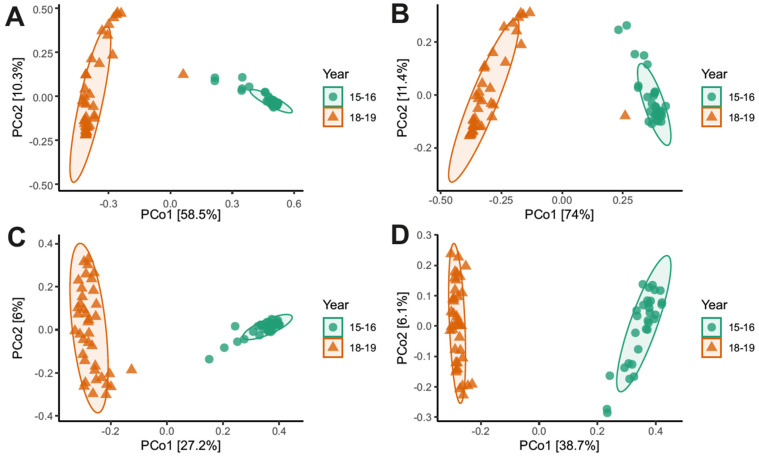
Principal Coordinate Analysis (PCoA) of bacterial community composition between the two sampling years (2015–2016 and 2018–2019) based on different beta diversity metrics: (**A**) Bray–Curtis, (**B**) Weighted UniFrac, (**C**) Jaccard, and (**D**) Unweighted UniFrac distances. Statistical differences between years were assessed using PERMANOVA (FDR-adjusted).

The beta diversity analyses revealed clear and significant differences in bacterial community composition between the two sampling years across all dissimilarity metrics (Figure 4A–D). The Bray–Curtis (Figure 4A) and Weighted UniFrac (Figure 4B) distances, which account for relative abundances and phylogenetic relationships, showed the highest degree of dissimilarity between years (R^2^ = 0.56 and 0.69; *p* = 0.001, FDR-adjusted). In contrast, the Jaccard (Figure 4C) and Unweighted UniFrac (Figure 4D) metrics, based on presence/absence data, displayed lower but still significant separation (R^2^ = 0.27 and 0.38; *p* = 0.001). All PERMANOVA results were adjusted using the false discovery rate (FDR) method and are reported in the Supplementary Materials Table S1. This model suggests that the interannual differences observed are mainly associated with changes in the relative abundance of dominant taxa rather than with major taxonomic or phylogenetic changes. Such results suggest that bacterial community structure at Concordia is largely influenced by stochastic processes and transient environmental inputs rather than by persistent deterministic selection.

### 3.4. Beta Dispersion

**Figure 5:**
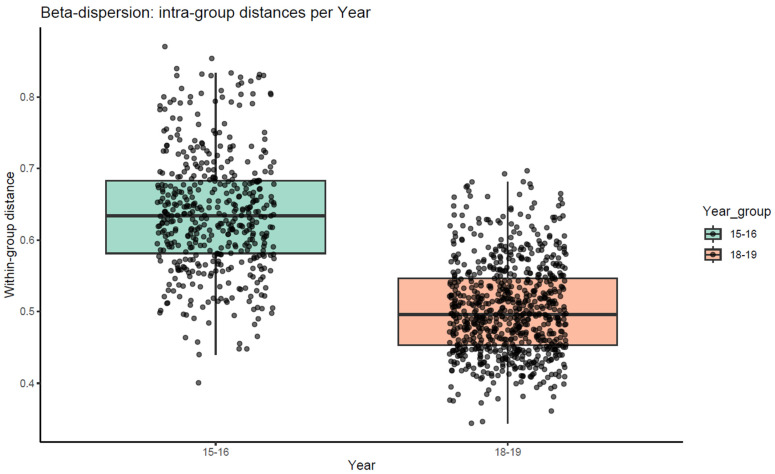
Comparison of beta dispersion of bacterial communities between the two sampling years (2015–2016 and 2018–2019). Dispersion values were calculated within groups by year using the cal_group_distance() function in *microeco*. Statistical support was tested by the Kruskal–Wallis rank sum test.

Beta dispersion analysis, calculated within groups by sampling year, revealed significant differences in community variability between the two datasets (Figure 5). Dispersion was higher in 2015–2016 (median = 0.63) than in 2018–2019 (median = 0.50), as confirmed by the Kruskal–Wallis test (*p* = 7.45 × 10^−121^, ****). These results indicate different levels of intra-annual heterogeneity between the two sampling periods.

### 3.5. Raup-Crick

**Figure 6:**
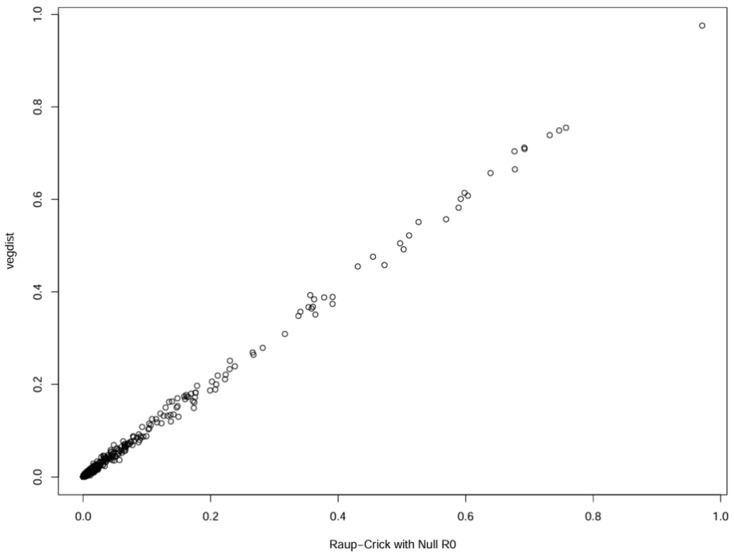
Comparison between observed and simulated bacterial community dissimilarities under the Raup-Crick R0 null model. The majority of sample pairs cluster near the 1:1 line with most points concentrated near zero deviation, indicating a strong stochastic component in community turnover.

To evaluate whether bacterial community assembly deviated from random expectations, a Raup-Crick null model analysis (R0) was performed using the *ecosimu()* function in the *vegan* package (Figure 6). In this model, community composition was randomised without constraining species richness, allowing for a comparison between observed and purely random assemblages. The relationship between observed and simulated Jaccard dissimilarities showed that most sample pairs were distributed close to the 1:1 line (Table S1), with the majority of points concentrated near zero deviation. This pattern indicates that the observed bacterial turnover between samples does not substantially differ from random expectations. This model indicates that the bacterial turnover observed among the samples does not deviate substantially from random expectations under the applied null model in the ice and snow around Concordia Station.

## 4. Discussion

The pronounced interannual variability observed in bacterial assemblages at Concordia Station suggests that the composition of the microbial community in this oligotrophic and extreme environment changes significantly over time. In the absence of stable sources of nutrients, persistent liquid water or continuous biological inputs, microbial persistence on the Antarctic Plateau is severely limited and community structure is likely to be influenced by episodic deposition and short-term survival rather than sustained local growth, competition or long-term establishment. Under these conditions, the microbial assemblages detected in surface snow are unlikely to represent fully structured ecological communities; instead, they are transient collections of propagules deposited from external sources. Under these conditions, the structure of the microbial community is expected to be weakly influenced by environmental filtering and strongly affected by dispersal-related processes, as predicted by neutral and stochastic models of microbial community assembly in low-biomass systems [10,11]. Similar patterns of stochastic assembly have been reported in other extremely oligotrophic environments, where microbial biomass is close to the detection limit and local growth is severely restricted. The combination of comparable richness but contrasting evenness across years, alongside pronounced interannual differences in beta diversity, suggests that bacterial communities in the vicinity of Concordia Station are not sustained by stable local niches. Instead, they appear to fluctuate in composition based on temporally variable inputs potentially associated with atmospheric transport, sporadic human activity or other episodic processes. Prior aerobiological research has revealed that long-range atmospheric transport is a primary mechanism through which microbes disperse to the Antarctic Plateau [1,25]. Indeed, several studies have demonstrated that microorganisms can be transported over intercontinental distances via high-altitude air masses, which can then deposit them in polar snowpacks [26,27,28]. Analyses of air mass trajectories suggest that Dome C is periodically affected by air flows originating from the sea, coast and continent, which are capable of transporting microorganisms over large spatial scales. Studies have shown that airborne microbial communities exhibit strong temporal variability, which is associated with atmospheric circulation patterns and source regions. This suggests that shifts in the origin of air masses may significantly influence the composition of deposited microbial assemblages [29]. These inputs are inherently variable in terms of both timing and taxonomic composition, which provides a plausible explanation for the pronounced turnover observed between sampling years. Although limited compared to other Antarctic stations, the human presence at Concordia may represent an additional episodic source of microbial input. Scientific and logistical activities, particularly during the Antarctic summer, involve maintenance operations and the prolonged occupation of confined spaces by humans. This can release microorganisms that are subsequently dispersed through ventilation systems or local wind circulation. Studies conducted at other polar research stations have shown that even minimal human activity can alter the composition of the airborne microbial communities in isolated and oligotrophic environments [8,25]. However, the severe environmental conditions that characterise the Antarctic Plateau, such as persistently low temperatures, intense UV radiation and extreme desiccation, are likely to severely limit microbial survival and metabolic activity, thereby reducing the persistence of any anthropogenic signal. At Dome C, surface temperatures often stay below −25 °C, even in summer, creating conditions that severely restrict metabolic activity and prevent active microbial proliferation. Consequently, any microorganisms detected in snow samples are more likely to be dormant or inactive cells than actively growing populations. Raup-Crick null model analysis also indicates that observed patterns of community dissimilarity do not significantly deviate from random expectations under the applied null hypothesis. This suggests that, in the absence of strong and persistent environmental filters, the turnover of the bacterial community at Concordia is consistent with assembly processes dominated by dispersal, ecological drift and random colonisation-extinction dynamics rather than by deterministic selection alone. Such stochastic assembly patterns are commonly reported in microbial ecosystems where immigration rates exceed local growth rates, resulting in assemblages that are largely shaped by dispersal rather than competitive interactions. The differences observed in beta dispersion between years are consistent and indicate varying degrees of heterogeneity within each sampling period. This suggests differences in community spread rather than convergence towards a single stable configuration. Collectively, these results support the idea that the Antarctic Plateau passively accumulates microorganisms from diverse biogeographic sources, offering limited opportunities for sustainable colonisation or adaptation, and acts as a microbial reservoir. This interpretation is consistent with previous studies that have described Antarctic surface snow as a temporary sink for airborne microorganisms rather than a stable microbial habitat. While human activity at Concordia does not appear to leave a stable or lasting microbial footprint, the sensitivity of such low-biomass systems emphasises the importance of managing biological contamination carefully in environments that would otherwise be unable to support active microbial ecosystems. Future studies that integrate microbiological data with detailed atmospheric circulation models and high-frequency temporal sampling will be essential in further clarifying the relative contributions of dispersal, environmental constraints and episodic inputs to shaping microbial diversity on the Antarctic Plateau. These approaches will also help to establish whether the observed patterns are a general feature of inland Antarctic environments or are specific to the Concordia region.

## 5. Conclusions

This study highlights significant variability between years in the bacterial assemblages inhabiting the snow surface at Concordia Station, suggesting limited temporal stability in one of the most extreme and oligotrophic environments on Earth. The significant differences in community composition observed between sampling years, alongside the patterns revealed by null model analyses, suggest that bacterial assemblages on the Antarctic Plateau do not converge towards a stable or consistent structure. Rather, community composition appears to reflect the combined effects of sporadic inputs and severe environmental constraints that restrict sustained microbial growth and long-term colonisation. In such a cold, oligotrophic environment, local environmental filtering is likely to be limited. The observed patterns are therefore consistent with scenarios in which the random deposition and short-term persistence of airborne microorganisms play a significant role. Under these conditions, the microorganisms detected in surface snow are more likely to represent transient depositional assemblages than stable, metabolically active ecological communities. The absence of a detectable or persistent microbial footprint associated with the presence of humans at Concordia also suggests that introduced microorganisms are unlikely to establish stable populations under these environmental constraints. Nevertheless, the sensitivity of these systems with extremely low biomass highlights how even minimal biological inputs can temporarily influence environments that would otherwise be unable to support sustained microbial activity. The stochastic deposition of biological propagules resulting from unpredictable atmospheric transport events may therefore vary considerably from year to year. Analyses of surface snow and ice samples provide a snapshot of the microorganisms deposited over relatively short timescales, likely reflecting atmospheric inputs accumulated over a few months. This is because the annual precipitation at Concordia Station is only around 20 mm of water equivalent. These findings are valuable for preserving the pristine nature of Antarctica’s inland ecosystems and for improving our understanding of microbial dynamics in other environments characterised by extreme isolation and limited habitability. In this context, the Antarctic Plateau can be considered a highly sensitive microbial reservoir where microbial assemblages are transient and weakly structured and where variability is largely associated with atmospheric inputs and environmental severity. To better understand microbial dynamics in such ultra-oligotrophic systems and support the protection and management of environments particularly vulnerable to external biological contamination, integrative and long-term monitoring approaches combining microbiological data, atmospheric circulation analyses and high-resolution temporal sampling will be essential.

## Data Availability

Raw sequences are available on the NCBI SRA under the bioproject PRJNA991501 for Stoppiello et al. 2023 [9] and PRJNA634088 for Napoli et al. 2021 [8].

## Supplementary Materials

The following supporting information can be downloaded at: https://www.mdpi.com/article/10.3390/life16040596/s1, Table S1: Statistics. The statistical table is organised into four sheets. The first sheet contains the alpha diversity values and the comparisons among the indices used in this study; the second sheet reports the beta diversity (PCoA) analysis, showing the differences between years for each distance matrix employed; the third sheet presents the beta dispersion analysis; and the fourth sheet includes the Raup-Crick analysis.
